# Recent Trends of Summer Convective and Stratiform Precipitation in Mid-Eastern China

**DOI:** 10.1038/srep33044

**Published:** 2016-09-08

**Authors:** Yunfei Fu, Fengjiao Chen, Guosheng Liu, Yuanjian Yang, Renmin Yuan, Rui Li, Qi Liu, Yu Wang, Lei Zhong, Liang Sun

**Affiliations:** 1 School of Earth and Space Sciences, University of Science and Technology of China, Hefei, Anhui 230026, PR China; 2 State Key Laboratory of Severe Weather, Chinese Academy of Meteorological Sciences, Beijing, 100081, PR China; 3 Anhui Meteorological Information Centre, Anhui Institute of Meteorological Science, Hefei, Anhui 230031, PR China; 4 Department of Earth, Ocean and Atmospheric Science, Florida State University, Tallahassee, Florida; 5 Key Laboratory of Atmospheric Sciences and Satellite Remote Sensing of Anhui Province, Anhui Institute of Meteorological Sciences, Hefei, Anhui 230031, PR China

## Abstract

Many studies have reported on the trends of precipitation in Mid-Eastern China (EC). However, the trends of convective and stratiform precipitation are still unknown. Here, we examine the trends of summer convective and stratiform precipitation in EC from 2002 to 2012 on the basis of the TRMM observations. Results revealed that the rain frequency (RF) for both convective and stratiform precipitation increased in majority regions of Southern EC (SEC), but decreased in Northwest part of Northern EC (NEC). The decreasing rate of RF for stratiform precipitation in NEC is twice as much as that for convective precipitation, while the increase of convective precipitation in SEC is more evident than stratiform precipitation. The rain rate (RR) exhibited a decreasing trend in most portions of EC for both convective and stratiform precipitation. In SEC, neither PW nor WVT has good ability in explaining the precipitation variability. However, in NEC, PW is closely correlated to convective RF and WVT is more closely related to stratiform RF.

Precipitation is not only a critical process in global hydrologic cycle but also an important indicator of climate change. The changes of temporal-spatial patterns of precipitation may potentially cause severe droughts or flood hazards^1^,^2^,^3^,^4^, which are responsible for tremendous human casualties and economic losses^5^,^6^. Previous studies have revealed the trends of precipitation in China using rain gauge data. There is a clear increase in boreal-summer precipitation amount over Yangtze River basin, but a decrease in northern China^7^,^8^,^9^,^10^. A few studies have also examined the trends of “precipitation intensity”^11^,^12^ as defined by the daily precipitation amount (unit: mm/day), which is really the daily rain accumulation. This definition may cause overestimation of precipitation intensity to some extent. For example, drizzle lasting a long time may produce substantial rain amount. The trend of observed instantaneous rain rate (unit: mm/h), which is a direct indicator of precipitation intensity, however, has rarely been examined. Furthermore, because of the limitation of rain gauge for classifying rain types in previous studies, this is the first study examining the trend of precipitation separately for convective and stratiform types.

In addition, the rapid industrialization in China has unavoidably led to a dramatic increase of aerosols^13^, just as what developed countries have once experienced^14^. The impact of aerosols on cloud and precipitation, known as the aerosol indirect effect^15^,^16^, is one of the most challenging problems in climate research. Researchers have found the evidence of precipitation depression and intensifying in heavy aerosol conditions^17^,^18^,^19^,^20^,^21^. These discrepancies indicate that the impacts of aerosol on precipitation are complex, which motivated us to pose the question of how convective and stratiform precipitation changes in heavy aerosol conditions.

With the launch of the Tropical Rainfall Measuring Mission’s (TRMM) satellite in 1997, over 10-year’s rainfall data derived by its Precipitation Radar (PR) for stratiform and convective precipitation are now available^22^, which provides us a unique opportunity to explore their recent trends. In this study, using the TRMM PR dataset 2A25 (see Methods for data details), we examined the changes of summer (June, July, and August) convective and stratiform precipitation in EC (26–35°N, 113–122°E), including the trends of rain rate (RR) and rain frequency (RF) in the period 2002–2012.

## Results

**Figure 1:**
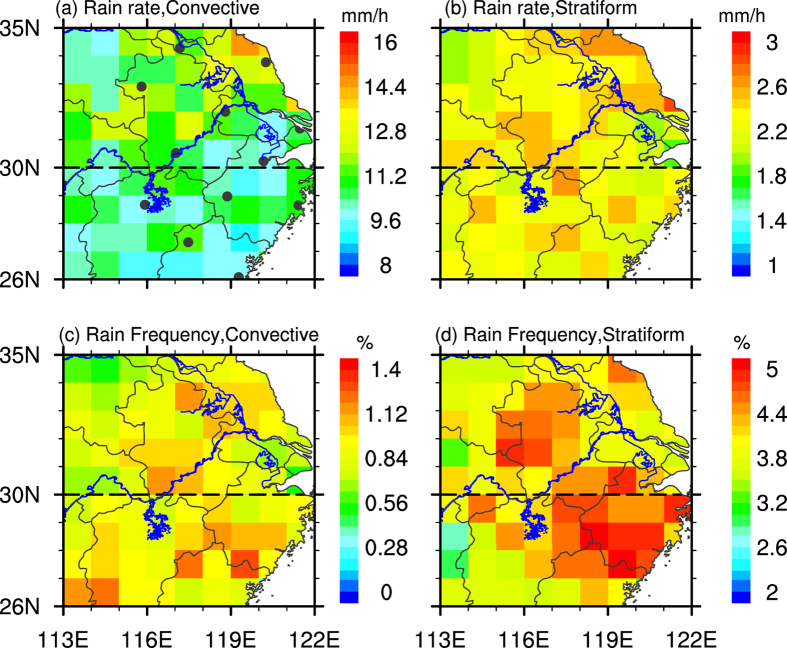
Spatial patterns of the summer mean rain rate (**a,b**) and rain frequency (**c,d**) for convective (left panel) and stratiform (right panel) precipitation at 1° × 1° resolution in EC during 2002–2012. Black dots in Figure 1a denote the locations of IGRA stations (see Methods). Maps were generated in NCAR Command Language (NCL)^42^.

Figure 1 displays the summer mean RR and RF of convective and stratiform precipitation at 1° × 1° resolution during 2002–2012. The summer mean RR for convective precipitation ranges from 9 mm/h to 14 mm/h, which is much larger than that for stratiform precipitation (1.8 mm/h~ 2.6 mm/h). And the mean RR in Northern EC (NEC, 30–35°N, 113–122°E) is generally larger than in Southern EC (SEC, 26–30°N,113–122°E) for both convective and stratiform precipitation. However, the summer stratiform RF varies from 3.2% to 5%, almost 3 times larger than that of convective precipitation (0.56%~1.4%), which is consistent with previous research^23^. Besides, regions of high convective and stratiform RF values locate along the Yangtze River, as well as part of SEC, which is probably associated with Asian summer monsoon^10^.

**Figure 2:**
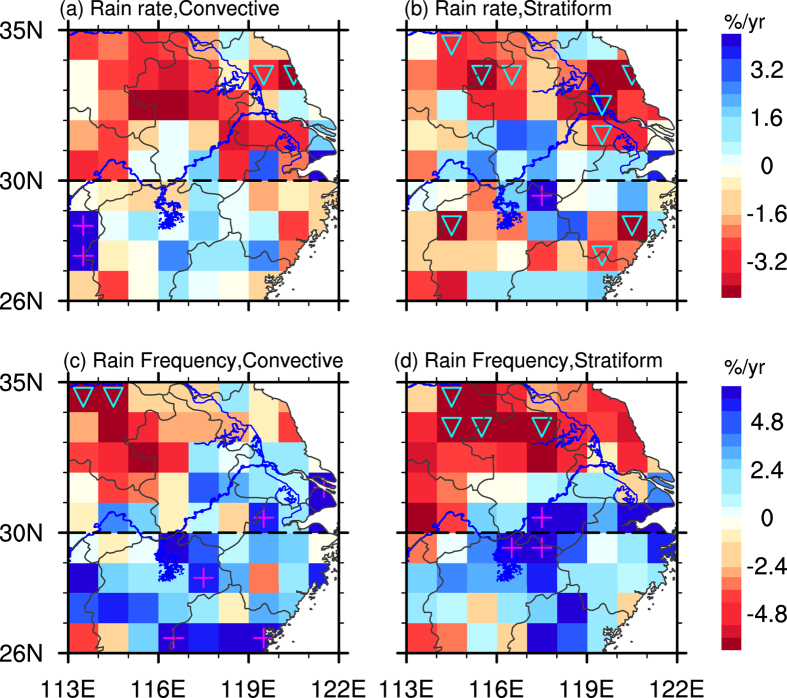
Spatial patterns of trends in normalized rain rate (**a,b**) and rain frequency(**c,d**) for convective (left panel) and stratiform (right panel) precipitation at 1° × 1° resolution over EC during summer of 2002 to 2012. Triangle/plus shows the negative/positive trends statistically significant at the 90% confidence level. Maps were generated in NCL^42^.

The trends in normalized anomalies (see Methods) of RR and RF for summer convective and stratiform precipitation during 2002–2012 are shown in Fig. 2. The normalized RR anomalies exhibit a clear decreasing trend in majority regions of NEC, reaching above 4.8% per year. In SEC, a relatively smaller increase trend is found for convective RR, but a much obvious decrease trend for stratiform precipitation. Considerable regional differences are found for the trends of convective and stratiform RF. For example, the RF for convective and stratiform precipitation has decreased by 2.4–4.8% per year in Northwest part of NEC, indicating that these regions have fewer precipitation events over the most recent 11 years. The downward trend is more significant for stratiform than for convective precipitation, with much more regions showing statistical significance at the 90% confidence level. In majority regions of SEC, positive trends are obtained for both convective and stratiform precipitation, but being more significant for convective precipitation. The trends of RF support the previous conclusion known as “South flood and North drought”^24^ pattern generated by rain gauges, but provide more details in rain types of convective and stratiform precipitation.

**Figure 3:**
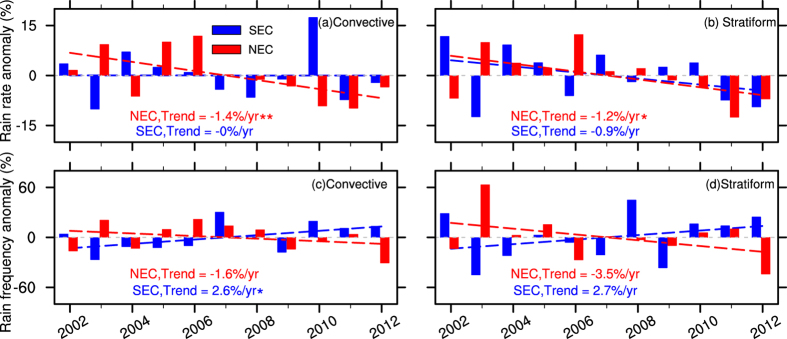
Area-averaged time series of annual normalized anomalies (%) of rain rate (**a,b**) and rain frequency (**c,d**) in SEC and NEC for convective (left panel) and stratiform (right panel) precipitation. Red (blue) dashed lines denote linear trends in NEC (SEC). ‘**’ and ‘*’ denote statistically significant at the 95% and 85% confidence level, respectively.

We further calculated the area-averaged time series of normalized anomalies of RR and RF in SEC and NEC for convective and stratiform precipitation. The results are presented in Fig. 3. Generally, the trends are similar to their corresponding spatial patterns (Fig. 2). The area-averaged RR exhibit a consistent decreasing trend in SEC and NEC for both convective and stratiform precipitation. In NEC, the decreasing trends of RR for convective and stratiform precipitation are much faster than those in SEC, reaching 1.4%/year (confidence level 95%) and 1.2%/year (confidence level 85%), respectively. In contrast, the trends of area-averaged RF show considerable regional differences. In NEC, the decreasing trend amplitude of stratiform RF reaches 3.5%/year, being approximately twice as much as that of convective precipitation (1.6%/year). In SEC, the RF increase of convective and stratiform precipitation reaches 2.6%/year and 2.7%/year, respectively, being much more significant (confidence level 85%) for convective precipitation.

**Figure 4:**
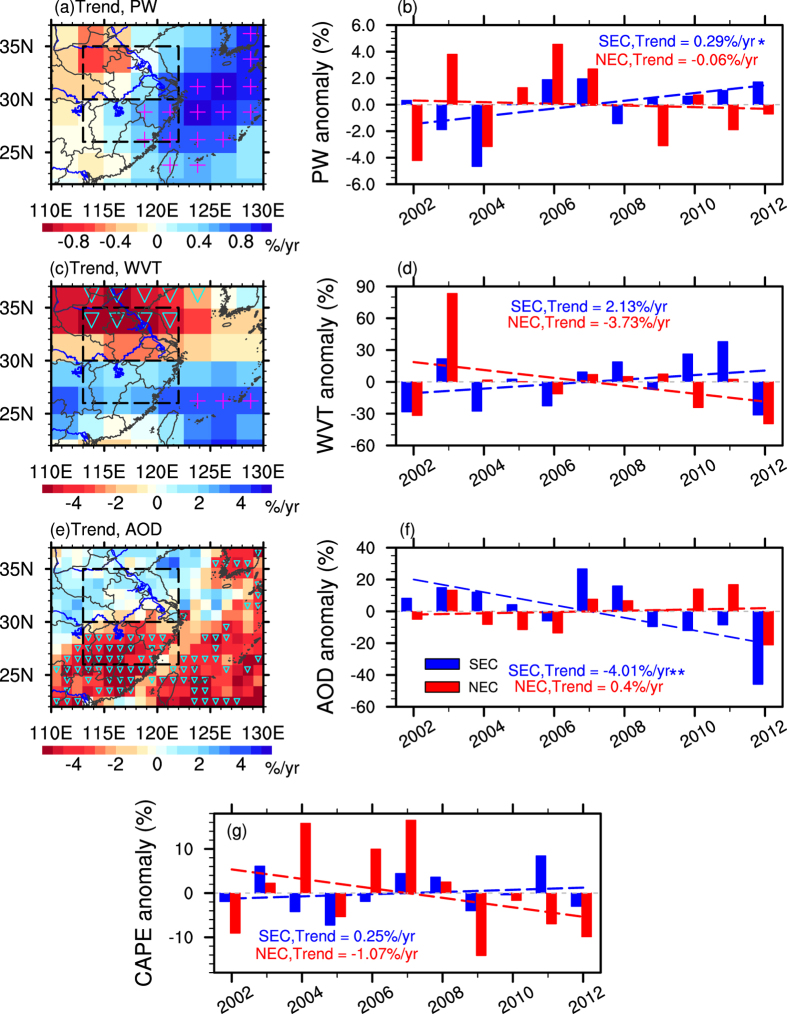
The spatial pattern of trends in normalized anomalies of PW (2.5° resolution) (**a**), WVT from 700 hPa to 500 hPa (2.5° resolution) (**b**), and AOD (1° resolution) (**e**) and the area-averaged time series of annual normalized anomalies (%) of PW (**b**), WVT in the 700 hPa to 500 hPa layer (**d**), AOD (**f**), and CAPE (**g**). Triangle/plus shows the negative/positive trends statistically significant at the 90% confidence level. Red (blue) dashed lines denote linear trends in NEC (SEC). ‘**’ and ‘*’ denote statistically significant at the 95% and 85% confidence level, respectively. Maps were generated in in NCL^42^.

The above results have shown considerable regional differences for the precipitation trends. We related precipitation to other climate variables^25^,^26^ to study the possible linkage between precipitation and climate variables. Here we firstly explored the trends of summer mean precipitable water (PW, see Methods) and water vapor transport (WVT, see Methods), presented in Fig. 4a–d. PW is the column integrated water vapor, a necessary ingredient for precipitation. The maximum positive trend of PW amounts to above 0.8% per year (significant at 90% confidence level), mainly located in East China Sea and coastal EC. The most distinct negative trend occurs in the northwestern part of NEC, reaching to 0.8% per year. The time series of area-averaged PW also show an upward trend in SEC and a smaller downward trend in NEC, in coincidence with its spatial patterns. The increase (decrease) of PW in SEC (NEC) is in good agreement with the change of precipitation, probably indicating a close linkage between PW and precipitation.

The WVT, representing the vertically integrated atmospheric water vapor transport from the adjacent regions, is one of the most important components in the East Asian monsoon system, which is clearly associated with precipitation process. Because of most water vapor being in the lower atmosphere, and also considering the relatively stable direction of moisture flux (southwest, which brings abundant moisture from the Arabian Sea and the Bay of Bengal^27^) in the layer between 700 hPa and 500 hPa in summer EC during 2002–2012 (the figure of annual WVT in this layer is omitted), the trends of the integrated moisture flux in the 700 hPa to 500 hPa layer (hereafter WVT) and its area-averaged time series are calculated, and displayed in Fig. 4c,d. A statistically significant decrease (confidence level 90%) trend of integrated moisture flux can be found in North part of NEC, ranging 2–4% per year. However, integrated moisture flux in SEC shows a smaller increase trend of 0–2%, indicative of more water vapor transport from Arabian Sea and the Bay of Bengal to SEC. For each region as whole, the mean integrated moisture flux exhibits similar positive trend of 2.13% per year in SEC and negative trend of 3.73% per year in NEC, respectively.

The aerosol conditions are also different in SEC and NEC. The mean aerosol optical depth (AOD; data taken from the MODIS^28^) ranges from 0.3 to 0.9 in summer EC (figure omitted). Heavy aerosol loadings with AOD exceeding 0.6 are found in NEC, whereas it is relatively clean in SEC. According to above results of different trends of precipitation in SEC and NEC, we can see that the regions in heavy aerosol conditions (i.e., NEC) exhibit downward trends for RR and RF for both rain types, especially for stratiform precipitation. Many previous studies have shown that aerosols have great impact on formation of clouds and precipitation^29^,^30^. Therefore, we further examined the trend of normalized anomalies of AOD, displayed in Fig. 4e,f. It is clear that the normalized AOD exhibits a statically significant (confidence level 90%) downward trend in SEC, reaching to above 4% per year (Fig. 4e,f). However, in NEC, a relatively smaller upward trend (~2%/year, not statistically significant) is found, indicating a relatively increasing aerosol loading over the most recent 11 years.

Previous study has found the evidence of aerosols’ suppression on precipitation by increasing the atmospheric stability^31^. To explore the possible linkage between aerosol and precipitation in EC, we further explored the trends of normalized anomalies of Convective Available Potential Energy (CAPE, see Methods) from Integrated Global Radiosonde Archive (IGRA, see Methods), displayed in Fig. 4g. The area-averaged CAPE exhibit a relatively clear positive trend in SEC and negative trend in NEC, suggesting the atmosphere becomes more stable in NEC and more instable in SEC over recent 11 years. According to previous studies^31^,^32^, aerosols can absorb the sunlight, heating the air and then increase the atmospheric stability, leading to the reduction of precipitation, known as the “positive feedback cycle”. Our results are in consistency with their conclusions. The heavy aerosol loadings are one of the possible mechanisms for the reduction of precipitation in NEC. More case studies and modeling studies are needed to be established to make our conclusion robust.

In order to make quantitative estimates on the relationships between PW (WVT) and precipitation, linear correlation coefficients (*R*) between area-averaged normalized anomalies of PW (WVT) and RR and RF are calculated, and listed in Table 1. As expected, RF is positively correlated with PW for convective and stratiform precipitation in SEC and NEC. That is, the greater the PW, the more frequent precipitation events. In addition, the correlation coefficient for convective RF is higher than that for stratiform precipitation in the same region, reaching 0.79 (significant at 90% confidence level) for convective RF. Although correlation does not imply causality, considering their physical connections, it is plausible to speculate that the positive (negative) trend of PW in SEC (NEC) may be one possible factor for the increase (decrease) of RF in SEC (NEC). In general, PW exhibits better correlations with precipitation (including RR and RF) in NEC than in SEC, probably because of the more complicated precipitation systems in SEC which results in a poor correlation of precipitation with a single atmospheric factor. Above results indicate a much closer correlation between regional PW and convective RF.

Similar to the positive relationships with PW and RF, WVT generally exhibits a positive relationship with RF. In addition, the correlation between WVT and RF in NEC is also much better than that in SEC, with a majority surpassing the 90% confidence level, where the maximum coefficient reaches 0.87 for stratiform RF in NEC. WVT is more closely linked to RF for stratiform precipitation (coefficients: 0.87) in NEC than convective precipitation (coefficients: 0.61), which probably indicates a great impact of water vapor transport on forming stratiform precipitation in NEC.

To quantify the relative importance of PW and WVT to the precipitation variability in SEC and NEC, we introduced coefficients of determinations (R^2^), which quantify the fractions of these variables that can explain the variation of precipitation. In SEC, the *R*^2^ values between PW and RR/RF, and between WVT and RR/RF are all small (the smallest value is 0.0025% (0.005^2^)), which indicates neither PW nor WVT being able to explain the variation of precipitation in SEC. PW can only explain up to 28% (0.53^2^) of convective RF variability in SEC, which is the largest *R*^2^ value in SEC. In NEC, *R*^2^ values are relatively large. For example, the WVT can explain up to 76% (0.87^2^) of stratiform RF variability. Meanwhile, PW can explain up to 62% (0.79^2^) of the convective RF variability in NEC. In conclusion, in NEC, water vapor transport is in much closer linkage with stratiform precipitation, while PW is in better relations with convective RF than WVT.

## Discussion

This research for the first time reveals the trends of convective and stratiform precipitation in EC over the most recent 11 years. The RF increased significantly (confidence level 90%) for majority regions of SEC and decreased significantly (confidence level 90%) in Northwestern NEC for both convective and stratiform precipitation. In heavy aerosol conditions (NEC), the reduction of stratiform precipitation is more evident than convective precipitation. The increase (decrease) of WVT and PW in SEC (NEC) is in good agreement with the positive (negative) trends of convective and stratiform RF. However, in SEC, neither PW nor WVT has good ability for explaining the precipitation variability. In NEC, PW is in much stronger correlation with convective precipitation, whereas WVT integrated from 700 hPa to 500 hPa is strongly correlated with stratiform precipitation. The heavy aerosol loadings tend to increase the atmospheric stability, it might be one of the possible mechanisms for the reduction of precipitation in NEC.

## Methods

The rainfall data used in this study is the TRMM PR 2A25 version 7 (hereafter referred to as PR 2A25) dataset, provided by GSFC/NASA (Goddard Space Flight Center, National Aeronautics and Space Administration). Precipitation information, including location in longitude and latitude, precipitation types (stratiform, convective and “others”) and near surface rain rate (unit: mm/h) etc., are given in each PR profile^22^,^33^. The horizontal resolution for this dataset is 4.3 km and decrease to 5.0 km after TRMM orbit boost. The PR 2A25 is the first rainfall dataset containing convective and stratiform precipitation in a relatively long time scale. The reliability and homogeneity of this precipitation dataset are of great importance to this study. A few studies have verified that PR 2A25 (version 7) is reliable and the biases are largely diminished, after comparing it with rain gauge and earlier version 6 data^34^. Due to the TRMM orbit boost in 2001, a few observation parameters (radar sensitivity, horizontal resolution, swath width, sampling and etc.) are changed, leading to the inhomogeneity of RR and RF^35^,^36^,^37^,^38^. Therefore, the data before 2001 are excluded from this study, and our study of trends in convective and stratiform precipitation is limited to the time period from 2002 to 2012.

In our analysis, the trends of summer precipitation, including RR and RF are fully investigated in EC during 2002–2012. Since the source 2A25 datasets are pixel datasets in irregular locations, it is inconvenient to study the trends based on the pixel resolution. To solve this problem, we construct a gridded dataset to present the spatial pattern of precipitation characteristics in this study. The RR is calculated from total rain rate (from PR 2A25) divided by the total rainy samples in each 1° longitude-latitude grid box, which is actually the mean rain rate in each grid. Because the sensitivity of PR is about 18 dBZ (post-boost), which corresponds to a rain rate of approximately 0.4 mm/h^39^, a rainy event is defined as an event with near surface rain rate larger than 0.4 mm/h. The RF is estimated by the ratio of the number of rainy events to the total PR measured samples (consists of rainy and non-rainy events). Ultimately, we obtain gridded datasets for two variables (RR and RF) at 1° longitude-latitude resolution.

Previous study^23^ has shown that the mean RR (RF) of convective precipitation is much larger (smaller) than that of stratiform precipitation. To make the magnitudes of interannual variations comparable between convective and stratiform precipitation, we introduce an index of normalized precipitation anomalies (*R*_*a*_) defined by the annual summer mean precipitation anomalies divided by the mean precipitation during the summers of 2002–2012 (formula 1).


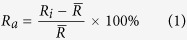


where *R*_*i*_ is the annual summer mean RR (RF), 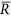 is the mean RR (RF) during the summers of 2002–2012. In the end, a simple linear regression method is used to estimate the trends of normalized precipitation anomalies. The trend is then statistically analyzed by t-test method.

The summer mean and trends of AOD are derived using the level-3 monthly aerosol products at 1° resolution from MODIS^28^ during 2002~2012. PW and WVT are estimated by specific humidity and wind vector from monthly NCEP/NCAR reanalysis data^40^ at 2.5° resolution, which reflect the water vapor content and atmospheric moisture transport, respectively. They are calculated with the following expressions:


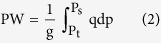



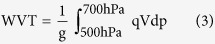


where P_s_ is the surface pressure, P_t_ is the top atmospheric pressure, q is the specific humidity, g equals to 9.8 m/s^2^, and V is the wind vector. Because the water vapor is nearly negligible above 300 hPa, the vertical integration of equation (2) is calculated from the surface to 300 hPa.

Atmospheric instability is illustrated by CAPE calculated from the IGRA^41^. This radiosonde dataset is provided by the National Climatic Data Center (http://www.ncdc.noaa.gov/data-access/weather-balloon/integrated-global-radiosonde-archive), which include meteorologic elements, such as pressure, temperature, geopotential height, dew point depression, and etc. 12 sounding stations are used in this study (presented in Fig. 1a), where 7 stations are located in NEC and 5 stations are located in SEC.

CAPE is the maximum energy available to an ascending parcel, calculated with the following expression:


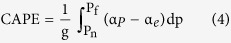


where P_f_ is the pressure at the level of free convection, P_n_ is the pressure at the level of neutral buoyancy, α_p_ is the specific volume of a parcel moving upward moist-adiabatically from the level of free convection, α_*e*_ is the environmental specific volume profile.

In addition, the oceanic regions of SEC and NEC are eliminated from precipitation analysis using the digital terrain data from National Geophysical Data Center (NGDC).

## Additional Information

**How to cite this article**: Fu, Y. *et al*. Recent Trends of Summer Convective and Stratiform Precipitation in Mid-Eastern China. *Sci. Rep.* **6**, 33044; doi: 10.1038/srep33044 (2016).

## Figures and Tables

**Table 1:** The linear correlation coefficients between normalized anomalies of PW/WVT and RR/RF in summer SEC and NEC during 2002 to 2012.

Rain Type	Precipitation	PW		WVT	
		SEC	NEC	SEC	NEC
convective	Rain rate	−0.07	0.63*	−0.23	0.37
	Rain Frequency	0.53*	0.79*	0.17	0.61*
stratiform	Rain rate	−0.21	0.68*	−0.30	0.53*
	Rain Frequency	0.23	0.29	0.005	0.87*

“*” denote statistically significant at the 90% confidence level

## References

[b1] Zong Y. & Chen X. The 1998 flood on the Yangtze, China. Natural Hazards **22**, 165–184 (2000).

[b2] Xin X., Yu R., Zhou T. & Wang B. Drought in late spring of South China in recent decades. Journal of Climate 19, 3197–3206 (2006).

[b3] Jiang T., Kundzewicz Z. W. & Su B. Changes in monthly precipitation and flood hazard in the Yangtze River Basin, China. International Journal of Climatology 28, 1471–1481 (2008).

[b4] Gemmer M. . Seasonal precipitation changes in the wet season and their influence on flood/drought hazards in the Yangtze River Basin, China. Quaternary International 186, 12–21 (2008).

[b5] Huang X.. Flood hazard in Hunan province of China: an economic loss analysis. Natural Hazards 47, 65–73 (2008).

[b6] Yi C. S., Lee J. H. & Shim M. P. GIS-based distributed technique for assessing economic loss from flood damage: pre-feasibility study for the Anyang Stream Basin in Korea. Natural hazards 55, 251–272 (2010).

[b7] Hu Z. Z., Yang S. & Wu R. Long-term climate variations in China and global warming signals, J. Geophys. Res. 108, 4614 (2003).

[b8] Yang F. L. & Lau K. M. Trend and variability of China precipitation in spring and summer: linkage to sea-surface temperatures. International journal of climatology 24, 1625–1644 (2004).

[b9] Ding Y. H. . Detection, Causes and Projection of Climate Change over China: An overview of recent progress. Advances in Atmospheric Sciences 24, 954–971 (2007).

[b10] Ding Y. H., Wang Z. Y. & Sun Y. Inter-decadal variation of the summer precipitation in East China and its association with decreasing Asian summer monsoon. Part I: Observed evidences. International Journal of Climatology 28, 1139–1161 (2008).

[b11] Karl T. R. & Knight R. W. Secular trends of precipitation amount, frequency, and intensity in the United States. Bull. Am. Meteorol. Soc. 79, 231–241(1998).

[b12] Liu B. H., Xu M., Henderson M. & Qi Y. Observed trends of precipitation amount, frequency, and intensity in China, 1960–2000. J. Geophys. Res. 110, D08103, doi: 10.1029/2004JD004864 (2005).

[b13] Tie X. . Chemical characterization of air pollution in eastern China and the eastern United States. Atmos. Environ. 40, 2607–2625 (2006).

[b14] Mayer H. Air pollution in cities. Atmos. Environ. 33, 4029–4037 (1999).

[b15] Twomey S. Pollution and the planetary albedo. Atmos. Environ. 8, 1251–1256 (1974).

[b16] Albrecht B. A. Aerosols, cloud microphysics, and fractional cloudiness. Science 245, 1227–1230 (1989). 17747885 10.1126/science.245.4923.1227

[b17] Rosenfeld D. Suppression of rain and snow by urban and industrial air pollution. Science 287, 1793–1796 (2000). 10710302 10.1126/science.287.5459.1793

[b18] Khain A., Rosenfeld D. & Pokrovsky A. Aerosol impact on the dynamics and microphysics of deep convective clouds. Q. J. R. Meteorol. Soc. 131, 2639–2663 (2005).

[b19] Min Q. L. . Evidence of mineral dust altering cloud microphysics and precipitation. Atmos. Chem. Phys. 9, 3223–3231 (2009).

[b20] Li Z. Q. . Long-term impacts of aerosols on the vertical development of clouds and precipitation. Nature Geoscience 4, 888–894 (2011).

[b21] Koren I. . Aerosol-induced intensification of rain from the tropics to the mid-latitudes. Nature Geoscience 5, 118–122 (2012).

[b22] Steiner M., Houze R. A. Jr & Yuter S. E. Climatological characterization of three-dimensional storm structure from operational radar and rain gauge data. Journal of Applied Meteorology 9, 1978–2007 (1995).

[b23] Liu P., Li C. Y., Wang Y. & Fu Y. F. Climatic characteristics of convective and stratiform precipitation over the Tropical and Subtropical areas as derived from TRMM PR. Science China. Earth Sciences 56, 375–385 (2013).

[b24] Gong D. Y. & Ho C. H. Shift in the summer rainfall over the Yangtze River valley in the late 1970s. Geophys. Res. Lett. 29, 78-1–78-4 (2002).

[b25] Lu D. R., Yang Y. J. & Fu Y. F. Interannual variability of summer monsoon convective and stratiform precipitations in East Asia during 1998–2013. Int. J. Climatol. doi: 10.1002/joc.4572 (2016).

[b26] Deng Y. Y., Gao T., Gao H. W., Yao X. H. & Xie L. Regional precipitation variability in East Asia related to climate and environmental factors during 1979–2012. Sci. Rep. 4, 5693; doi: 10.1038/srep05693 (2014). 25033387 PMC4102078

[b27] Zhou T. J. & Yu R. C. Atmospheric water vapor transport associated with typical anomalous summer rainfall patterns in China. Journal of Geophysical Research: Atmospheres 110 (2005).

[b28] Chu D. A. . Validation of MODIS aerosol optical depth retrieval over land. Geophys. Res. Lett. 29, MOD2-1–MOD2-4 (2002).

[b29] Twomey S. Aerosols, clouds and radiation. Atmos. Environ. 25, 2435–2442 (1991).

[b30] Rosenfeld D. Suppression of rain and snow by urban and industrial air pollution. Science 287, 1793–1796 (2000). 10710302 10.1126/science.287.5459.1793

[b31] Zhao C. S., Tie X. X. & Lin Y. P. A possible positive feedback of reduction of precipitation and increase in aerosols over eastern central China. Geophys. Res. Lett. 33, L11814 (2006).

[b32] Rosenfeld D. . Flood or drought: how do aerosols affect precipitation? Science 321 1309–1313 (2008). 18772428 10.1126/science.1160606

[b33] Awaka J., Iguchi T. & Okamoto K. TRMM PR standard algorithm 2A23 and its performance on bright band detection. J. Meteorol. Soc. Jpn. 87A, 31–52 (2009).

[b34] Barros A. P., Joshi M., Putkonen J. & Burbank D. W. A study of the 1999 monsoon rainfall in a mountainous region in central Nepal using TRMM products and rain gauge observations. Geophys. Res. Lett. 27, 3683–3686 (2000).

[b35] DeMoss J. D. & Bowman K. P. Changes in TRMM rainfall due to the orbit boost estimated from buoy rain gauge data. Journal of Atmospheric and Oceanic Technology 24, 1598–1607 (2007).

[b36] Shimizu Shuji . Evaluation of the effect of the orbit boost of the TRMM satellite on the PR rain estimates. Geoscience and Remote Sensing Symposium, IGARSS 2008. IEEE International. 4 (2008).

[b37] Nakazawa T. & Rajendran K. Interannual variability of tropical rainfall characteristics and the impact of the altitude boost from TRMM PR 3A25 data. J Meteorol Soc Jpn. 87, 317–338 (2009).

[b38] Liu X. T., Fu Y. F. & Liu Q. Significant impacts of the TRMM satellite orbit boost on climatological records of tropical precipitation. Chin. Sci. Bull. 57, 4627–4634 (2012).

[b39] Schumacher C. & Houze R. A. The TRMM Precipitation Radar’s View of Shallow, Isolated Rain. J. Appl. Meteorol. 42, 1519–1524 (2003).

[b40] Kalnay E. . The NCEP/NCAR 40-year reanalysis project. Bull. Amer. Meteor. Soc. 77, 437–470 (1996).

[b41] Durre I., Vos R. S. & Wuertz D. B. Overview of the integrated global radiosonde archive. J. Clim. 19, 53–68 (2006).

[b42] The NCAR Command Language (Version 6.1.0) [Software]. Boulder, Colorado: UCAR/NCAR/CISL/TDD. http://dx.doi.org/10.5065/D6WD3XH5 (2012).

